# Janus Self‐Propelled Chitosan‐Based Hydrogel Spheres for Rapid Bleeding Control

**DOI:** 10.1002/advs.202205989

**Published:** 2022-12-25

**Authors:** Qiao Yu, Baihai Su, Weifeng Zhao, Changsheng Zhao

**Affiliations:** ^1^ Department of Nephrology West China Hospital Sichuan University Chengdu 610041 China; ^2^ Institute for Disaster Management and Reconstruction Sichuan University Chengdu 610207 China; ^3^ Med‐X Center for Materials Sichuan University Chengdu 610041 China; ^4^ College of Polymer Science and Engineering State Key Laboratory of Polymer Materials Engineering Sichuan University Chengdu 610054 China

**Keywords:** chitosan, gravity settlement, hemorrhage, hemostasis, self‐propulsion

## Abstract

Uncontrolled hemorrhage is a major cause of potentially preventable death in civilian trauma nowadays. Considerable concern has been given to the development of efficient hemostats with high blood absorption, self‐propelled property, and Ca^2+^ release ability, for irregularly shaped and noncompressible hemorrhage. Herein, Janus self‐propelled chitosan‐based hydrogel with CaCO_3_ (J‐CMH@CaCO_3_) is developed by partial ionic crosslinking of carboxylated chitosan (CCS) and Ca^2+^, gravity settlement, and photopolymerization, followed by removing the shell of CCS. The obtained J‐CMH@CaCO_3_ is further used as a hemostat powered by the internal CaCO_3_ and coordinated protonated tranexamic acid (J‐CMH@CaCO_3_/T). Bubbles are generated and detached to provide the driving force, accompanied by the release of Ca^2+^. The two aspects work in synergy to accelerate clot formation, endowing the J‐CMH@CaCO_3_/T with excellent hemostatic efficiency. The J‐CMH@CaCO_3_/T presents high blood absorption, favorable blood‐clotting ability, desired erythrocyte and platelet aggregation, and acceptable hemocompatibility and cytocompatibility. In rodent and rabbit bleeding models, the J‐CMH@CaCO_3_/T exhibits the most effective hemostasis to the best knowledge of the authors, wherein the hemorrhage is rapidly halted within 39 s. It is believed that the J‐CMH@CaCO_3_/T with self‐propelled property opens up a new avenue to design high‐performance hemostats for clinical application.

## Graphical abstract

Janus self‐propelled chitosan‐based hydrogel with CaCO_3_ and tranexamic acid (J‐CMH@CaCO_3_/T) presents stable self‐propelled property and Ca^2+^ release ability, high blood absorption, favorable blood‐clotting ability, desired erythrocyte and platelet aggregation, and acceptable hemocompatibility and cytocompatibility for irregularly shaped and noncompressible hemostasis. Notably, a new strategy to construct hydrogel spheres with Janus structure is also mentioned.

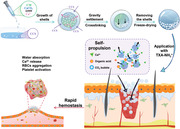

## 1 Introduction

External hemorrhage is a major cause of “potentially preventable” death in civilian trauma nowadays.^[^ ^1^ ^]^ Massive blood loss from junctional, truncal, and internal noncompressive injuries has been reported to result in significant pre‐hospital mortalities.^[^ ^2^ ^]^ Efficient hemostasis significantly alleviates suffering, prevents further injury, promotes wound recovery, and increases survival rate.^[^ ^3^, ^4^ ^]^ At present, pieces of gauze, tissue, and tourniquets are commonly used in prehospital care, which usually fail to achieve desired results in a timely manner since blood coagulation of most materials occurred only on the surface of the wounds. Also, they are not suitable for irregular, and noncompressible wounds^[^ ^5^ ^]^ due to the inherent structure.

To address these concerns, considerable improvements in the hemostatic dressings (in the form of sponges,^[^ ^6^ ^]^ sprayable foams,^[^ ^7^, ^8^ ^]^ in situ hydrogels,^[^ ^9^ ^]^ and powders/particles,^[^ ^10^ ^]^ etc.) with the capacity to coagulate blood at the bleeding sites have been developed. Expandable hemostats composed of sponges or cryogels^[^ ^11^ ^]^ have been delivered to the internal bleeding sites and then expanded to halt bleeding by recovering to their original shapes. However, overexpansion of these hemostats at internal bleeding sites may generate excessive pressure, leading to secondary hemorrhage. Similar to sponges, foams exert pressure on the wound surface by expanding in volume to stop bleeding. However, the foam may rapidly defoam and transform into a thin liquid,^[^ ^12^ ^]^ and the wounds re‐bleed immediately upon foam removal.^[^ ^13^ ^]^ Alternatively, in situ hydrogel has been developed to seal blood vessels by filling the irregular wounds through chemical reactions such as Schiff base couplings or enzymatic reactions.^[^ ^13^ ^]^ There is no denying that in situ hydrogels achieve hemostasis,^[^ ^14^ ^]^ but it needs time (commonly within 2 min) to transform to a gel state. Furthermore, to accelerate the gelation process, in situ hydrogels have been prepared within a second of UV exposure provided by a portable light source,^[^ ^15^ ^]^ but auxiliary equipment is not convenient for pre‐hospital care. Therefore, advanced hemostats are supposed to have minimal dimensions and flexible shapes to accommodate deep, irregular, noncompressible wounds and be easy to employ in pre‐hospital care.

Considering these requirements, hemostats based on particles have demonstrated possible applications for stopping hemorrhage in complex wounds. They can easily access bleeding sites due to their micro/nanosize. Strong blood flow from the wounds, however, may significantly impede their capacity for adaptation. Thus, it is an urgent need to design effective hemostatic agents^[^ ^16^ ^]^ with the ability to drive themselves against blood flow, which could reach the bleeding sites and fast hemorrhage process.^[^ ^17^ ^]^ Janus microspheres with gas bubbles generation are suggested as a promising method for directing themselves into underlying bleeding sites. For instance, Li et al.^[^ ^18^ ^]^ designed a potential hemostasis system by loading protonated tranexamic acid (TXA‐NH_3_^+^) with Janus hemostatic particles comprising microporous starch and CaCO_3_ crystals (Janus MSS@CaCO_3_). This hemostat is more effective than conventional hemostats, because a large number of floating bubbles are generated when the hemostat contact with blood that forces itself to move and pull into the deep hemorrhage sites. However, in the strategy, the storage and reaction conditions for *α*‐amylase and glucoamylase, the raw materials used in the starch enzymolysis process, are rigorous. The nucleation and growth process of CaCO_3_ crystals on the surface^[^ ^19^ ^]^ of starch require delicate control to obtain asymmetric structures for improving movement patterns. More effective easy‐to‐operate strategies are therefore required for the development of hemostats with asymmetric structure and self‐propelled ability.

The masking method is also one of the most general ways to prepare Janus particles.^[^ ^20^ ^]^ The uniform particles are fixed onto preprocessed glass slides, and then one side of the particles is functionalized by physical or electron beam deposition.^[^ ^21^ ^]^ This method requires precise control of the degree of submergence of the particles and is not suitable for large‐scale production. Also, Janus particles can be successfully fabricated by droplet microfluidics. The general procedure for this method is to first generate monodispersed droplets with microfluidic technology, then control the coalescence of two distinct droplets, and finally solidify the combined phases.^[^ ^22^ ^]^ However, both diffusive mixing and convective transport in microchannels must be taken into account.^[^ ^23^ ^]^

To solve the problems mentioned above, we recently developed a multifunctional asymmetric hydrogel through inertial settlement and asymmetrical distribution of particles within the hydrogel.^[^ ^24^ ^]^ Taking the advantage of forming spherical hydrogel shells using natural macromolecule CCS via ionic‐crosslinking in an alcohol‐aqueous binary solvent,^[^ ^25^ ^]^ it is possible to create hydrogel microspheres with an asymmetric structure for hemostasis by fusing inertial settlement and spherical hydrogel shells construction. In detail, we first reported the development of Janus self‐propelled chitosan‐based hydrogel with CaCO_3_ (J‐CMH@CaCO_3_) by partial ionic crosslinking of CCS and Ca^2+^, gravity settlement until the CaCO_3_ was unevenly distributed in spheres, and photopolymerization, followed by removing the CCS shell. The J‐CMH@CaCO_3_ was used as a hemostat powered by internal CaCO_3_ and coordinated TXA‐NH_3_^+^ (J‐CMH@CaCO_3_/T). The morphology, swelling property, and self‐propelled behavior of the J‐CMH@CaCO_3_ were investigated in detail. Moreover, the hemocompatibility and cytocompatibility were studied. The in vitro hemostatic property was assessed by blood clotting index (BCI) and erythrocyte aggregation. Finally, we examined the hemostatic performance of the J‐CMH@CaCO_3_/T in vivo through rodent bleeding models to provide references for clinical application.

## 2 Results and Discussion

### 2.1 Fabrication and Morphological Characterization

**Figure 1:**
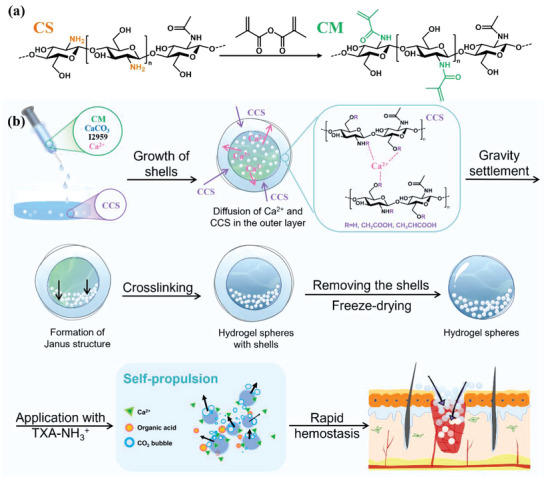
a) Scheme for the synthesis of CM. b) Schematic illustrations for the preparation and hemostatic application of the J‐CMH@CaCO_3_/T.

As shown in **Figure** **1**, a series of the hydrogel spheres with high water absorption, self‐propelled, and well‐defined Ca^2+^ release properties as hemostatic dressings were developed based on derivatives of chitosan. CM was synthesized by grafting methacrylic anhydride (MA) to the primary amine of CS molecule chains (Figure 1a). Unlike CS, the CM after lyophilization presented a cotton‐like fluffy appearance (Figure S1a, Supporting Information). Compared with CS, the FTIR spectrum of CM showed several new absorption peaks at 1654, 1546, and 1220 cm^−1^, which were attributed to amide I stretching, amide II bending, and amide III vibrations, respectively.^[^ ^26^, ^27^ ^]^ The absorption peak at 1617 cm^−1^ of CM was attributed to C=C stretching vibration, and overlapped with the peaks from the amide I.^[^ ^28^ ^]^ Moreover, the characteristic absorption peak at 1220 cm^−1^ of CS was attributed to the primary amine (—NH_2_) disappeared in CM (Figure S1b, Supporting Information). The thermal degradation of both curves of CS and CM showed two main steps of decomposition (Figure S2a, Supporting Information). The first decomposition at around 100 °C was due to the loss of water, and the weight loss in the second stage at around 300 °C was mainly due to the degradation of the chitosan molecules.^[^ ^29^ ^]^ Compared with CS, a new weight loss stage of CM was observed at 300–350 °C, indicating the successful introduction of the MA unit (Figure S2b, Supporting Information). Overall, the FTIR and TGA results indicated that the MA was covalently grafted to CS backbone through the acylation reaction.

J‐CMH@CaCO_3_/T was prepared via an easy‐to‐operate method, as schematically shown in Figure 1b. In the preparation of the J‐CMH@CaCO_3_, CM (substrate), I2959 (initiator), CaCO_3_ (self‐propulsion source), and CaCl_2_ and CCS (sacrificial mini‐reactors) were used. The J‐CMH@CaCO_3_/T was made up of J‐CMH@CaCO_3_ and TXA‐NH_3_^+^. Based on spherical hydrogel shell construction and gravity settlement, the J‐CMH@CaCO_3_/T was prepared through an ingenious reaction to benefit hemostasis. Besides, the hydrogel spheres only prepared by CM were named as CMH, while the hydrogel spheres that had CaCO_3_ without the gravity settlement step were also fabricated, and labeled as CMH@CaCO_3_.

**Figure 2:**
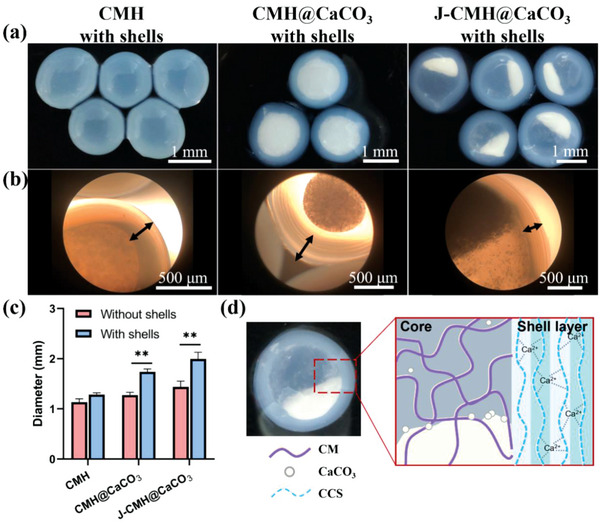
a) Digital images and b) optical microscopy images of the hydrogel spheres with shells. c) The average diameter of the hydrogel spheres with and without shells. d) Schematic for production of a hydrogel shell with a core.

The Ca^2+^ in the as‐prepared spinning solution is easily cross‐linked with CCS in the coagulation bath through the interaction between metallic ions and carboxyl groups.^[^ ^30^ ^]^ Therefore, when the Ca^2+^‐containing spinning solution was dropped into the coagulation bath, CCS diffused into the droplets to form the shells. The CMH with shells showed a homogeneous and transparent form (**Figure** **2**a). With the introduction of CaCO_3_ powder, the core of CMH@CaCO_3_ with shells became milky white. Also, the CaCO_3_ powder was unevenly distributed in the J‐CMH@CaCO_3_ with shells. In order to further explore the structure morphology, the hydrogel spheres with shells were investigated by optical microscope (Figure 2b). Interestingly, the shells appeared as concentric circles, similar to the annual rings of a tree. The concentric circles further supported the shells to form layer‐by‐layer along with the incubation time. The shells were used as sacrificial mini‐reactors to prepare Janus spherical hydrogels.^[^ ^25^ ^]^ The monomers in the shells were turned into spherical hydrogels quickly through photopolymerization under UV irradiation. It is notable that the monomers exhibited a liquidity characteristic. Depending on this property, the asymmetric structure of CaCO_3_ powder in the hydrogel spheres was easily generated by gravity settlement. The Janus structure of CaCO_3_ powder was properly fixed with a covalent crosslinking network after the polymerization under UV light. A little CaCO_3_ powder at the outer edges may escape with the dissolution of the shell, but large amount of CaCO_3_ powder remained in the hydrogel spheres and played a role in the subsequent hemostatic process. In addition, as shown in Figure 2c, the hydrogel spheres with shells exhibited a uniform size distribution with the average diameter of 1.28, 1.73, and 1.99 mm, respectively. The size of the hydrogel spheres with shells was controlled by the inner diameter of the syringe needle and/or the reaction time of the as‐prepared spinning solution bathing in the coagulation bath. Also, the voltage used in dropping process was closely related to the surface tension of the spinning solution, higher voltage led to a higher surface tension,^[^ ^31^ ^]^ forming a smaller diameter of hydrogel spheres with shells. Therefore, by controlling the parameters mentioned above, the hydrogel spheres with small diameter were synthesized. The diameter of the hydrogel spheres slightly increased from 1.13 mm to 1.44 mm along with the addition of CaCO_3_ powder after removing the shell. The increased hydrogel sphere size was attributed to the addition of CaCO_3_ powder into the spinning solution, which might affect the viscosity and conductivity of monomer solution during the spinning process and provide mechanical support for photocrosslinking.^[^ ^32^ ^]^ The schematic for the production of a hydrogel shell with a core of the J‐CMH@CaCO_3_ was illustrated in Figure 2d. That is, the diffusion of Ca^2+^ made the CCS form the shells, and they were used as the templates to produce the hydrogel spheres.

### 2.2 Water and Blood Absorption

**Figure 3:**
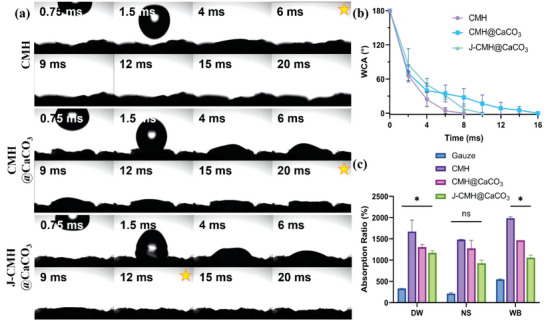
a) Images of WCAs of samples. Stars mean water has fully penetrated into the hydrogel spheres. b) Quantitative statistics of WCAs. c) Absorption ratios of samples in different liquids.

Owing to inherent porous structure and hydrophilicity, hydrogel‐based hemostatic agents would absorb a large amount of water from serum and wound exudate, concentrating coagulation factors, erythrocytes, and platelets.^[^ ^33^ ^]^ As observed in **Figure** **3**a, the WCAs of the CMH, CMH@CaCO_3_, and J‐CMH@CaCO_3_ decreased extremely quickly (in seconds, Movie S1, Supporting Information), suggesting that loose porous networks of the hydrogel resulted in quick swelling capacity. The CMH was able to absorb the water completely in 6 ms while the CMH@CaCO_3_ took a longer time. Affected by the distribution of CaCO_3_, the CMH exhibited better hygroscopicity than the CMH@CaCO_3_ in the same period (Figure 3b). The CMH and CMH@CaCO_3_ represented the different asymmetrical sides of the J‐CMH@CaCO_3_, respectively, indicating that an uneven rate of water absorption may promote its directional movement. The absorption ratios of the CMH, CMH@CaCO_3_ and J‐CMH@CaCO_3_ in DW were 1670%, 1300%, and 1170% (Figure 3c), respectively, which were remarkably larger than that of gauze. The absorption ratios showed a slight decrease in NS, because the salinity affected the degree of swelling of the hydrogel. The electrostatic repulsion between CM and Na^+^ made the localized expansions of hydrogel networks difficult.^[^ ^34^ ^]^ Notably, the higher absorption ratio in WB may be attributed to the accumulation of blood components inside the hydrogel spheres thereby inducing blood coagulation. In addition, the absorption ratios of the CMH@CaCO_3_ and J‐CMH@CaCO_3_ reduced slightly compared with that of the CMH, since the CaCO_3_ affected the inner pore structure of the hydrogel, but still maintained high absorption ratios.

### 2.3 Self‐Propelling Behavior

**Figure 4:**
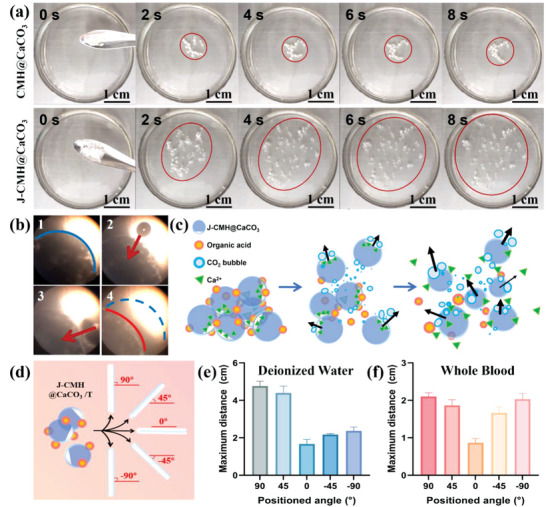
a) Images captured after a sprinkling of the CMH@CaCO_3_ and J‐CMH@CaCO_3_ into DW (pH = 4.3 with the addition of TXA‐NH_3_^+^ powder) in a horizontal direction. b) The corresponding optical micrographs of the J‐CMH@CaCO_3_/T moving in DW. c) Schematic representation of the possible motion behaviors showing the J‐CMH@CaCO_3_/T released CO_2_ and propelled themselves when placed in DW. d) Schematic showing displacement of the J‐CMH@CaCO_3_/T at different angles in glass tubes. The maximum self‐propelling distance in e) DW and f) WB.

CaCO_3_, as inorganic nanoparticle, can be dissolved in acidic condition, making it suitable as pH‐responsive material.^[^ ^35^ ^]^ TXA‐NH_3_^+^ is water‐soluble, and creates a transient acidic environment when it is dissolved in water. Thus, mixing CaCO_3_ with TXA‐NH_3_^+^ is capable of producing gas, promoting their movement in aqueous solutions. The CMH@CaCO_3_ and J‐CMH@CaCO_3_ displayed different motion behaviors (**Figure** **4**a, Movie S2, Supporting Information) in DW (pH = 4.3). Constant production of small bubbles was observed in the CMH@CaCO_3_. However, the homogeneous distribution of CaCO_3_ inside the CMH@CaCO_3_ might balance the forces among the bubbles, showing insignificant movement. On the contrary, the J‐CMH@CaCO_3_ offered a remarkable insight into horizontal diffusion, which generated bubbles rapidly and spread laterally at the surface in a horizontal direction within seconds. A considerable number of CMH@CaCO_3_ might stay on the superficial layers of the wound due to the generation of the nondirection bubble, leading to a poor hemostatic effect. Thus, J‐CMH@CaCO_3_ could improve the movement pattern by Janus structure. The forces among the bubbles could apply over the hydrogel spheres and pull them move into the bleeding sites, then improve hemostatic efficiency.

An optical microscope was also used to elucidate the motion behaviors of the J‐CMH@CaCO_3_/T (Figure 4b, Movie S3, Supporting Information). The generation and detachment of bubbles were observed in the images. When the J‐CMH@CaCO_3_/T contacted water, bubbles were generated and then detached to provide the driving force. As shown in Figure 4c, the self‐propulsion ability of the J‐CMH@CaCO_3_/T was powered by the internal CaCO_3_ and coordinated TXA‐NH_3_^+^. After placing in water, the CaCO_3_ in the J‐CMH@CaCO_3_/T propelled the hydrogel spheres through the decomposition under acidic conditions, followed by bubbles formation. The self‐propulsion property of the J‐CMH@CaCO_3_/T was further determined by evaluating the maximum self‐propelling distance in DW and WB at different angles (Figure 4d). As shown in Figure 4e, when the J‐CMH@CaCO_3_/T was fed horizontally (0°), the maximum distance was 1.66 cm. The detachment force of the bubbles was mostly responsible for the motion. After adjusting the angle, the maximum distance increased by 1.3‐2.8 times, which might be attributed to the influence of buoyancy or gravity, driving the J‐CMH@CaCO_3_ to move upward or downward, respectively. Since the angle was set to 90°, the detached CO_2_ bubbles affected the motion of the J‐CMH@CaCO_3_, and the buoyancy following decomposition of CaCO_3_ inside the J‐CMH@CaCO_3_ was impacted as follows. Moreover, when the angle was set to −90°, gravity took priority and the bubble detachment acted as a secondary force to assist in propelling downward. The maximum propelling distance was slightly decreased in whole blood (Figure 4f). Notably, the blood clots adhering to the J‐CMH@CaCO_3_ were able to increase gravity and promote downward movement. Therefore, the J‐CMH@ CaCO_3_/T exhibited good self‐propelling property against DW and WB in different directions and could drive into noncompressible and irregular wound sites, ensuring effective hemorrhage control.

### 2.4 In Vitro Hemostatic Assay

**Figure 5:**
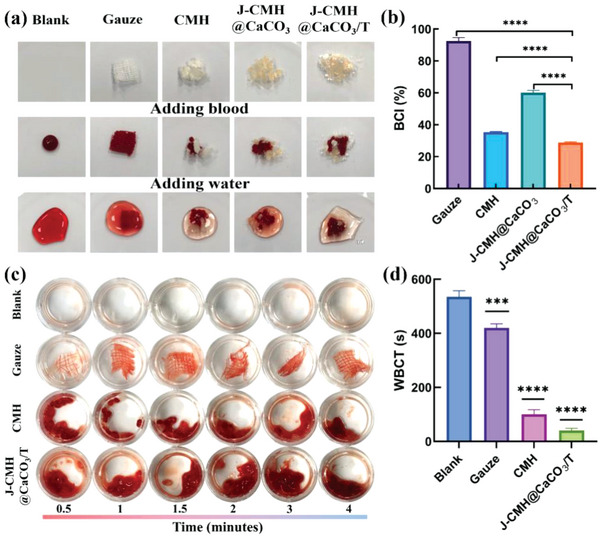
a) Optical images showing blood coagulation induced by different samples. b) A quantitative analysis of BCI. c) Images of WBCT treated with samples. d) A quantitative analysis of WBCT. The untreated blood was served as control, labeled as blank.

The hemostatic property can be reflected by optical images and BCI. The in vitro BCI test is a common method to assess the pro‐coagulation capacity of hemostatic materials.^[^ ^36^ ^]^ A higher BCI indicates a slower clotting rate.^[^ ^37^ ^]^ As a traditional hemostatic agent, gauze was used as a control. As shown in **Figure** **5**a, the blood diffusion in water was clearly visible in the blank and gauze groups, showing poor hemostasis. However, the water in the hydrogel spheres remained relatively clear, indicating a greater ability to coagulate blood. The BCI of the samples after filtration was calculated and shown in Figure 5b. The BCI values of the hydrogel spheres groups (the CMH, J‐CMH@CaCO_3_, J‐CMH@CaCO_3_/T) were significantly lower than that of the gauze group. Interestingly, the J‐CMH@CaCO_3_ showed lower BCI than the CMH, since the presence of CaCO_3_ powder causes the inability to absorb and aggregate blood components,^[^ ^38^ ^]^ which might slightly reduce the blood‐clotting property. In addition, the introduction of TXA‐NH_3_^+^ decreased the BCI by promoting Ca^2+^ release within the spheres, giving the J‐CMH@CaCO_3_/T with the best hemostatic property. The whole blood clotting time (WBCT) was investigated to explore the blood coagulation action of blank, gauze, CMH, and the J‐CMH@CaCO_3_/T on whole blood (Figure 5c). The collected blood clot associated with the J‐CMH@CaCO_3_/T was observed to be darker than those with the CMH and medical gauze in the same period. The darker red shown in the clot was attributed to the blood clots made up of blood cells and fibrins trapped by the J‐CMH@CaCO_3_/T. Meanwhile, WBCT was calculated for 3 groups at least and presented in Figure 5d. The CMH and J‐CMH@CaCO_3_/T significantly reduced the blood clotting time compared to gauze (420 s) and blank sample (535 s). Among all the samples, the J‐CMH@CaCO_3_/T exhibited efficient procoagulant activity (40 s). Activated partial thromboplastin time (APTT), prothrombin time (PT), and thrombin time (TT) were used to analyze coagulation pathways (endogenous, exogenous, and common pathways).^[^ ^39^ ^]^ As summarized in Table S3 (Supporting Information), both the CMH and J‐CMH@CaCO_3_/T could significantly shorten APTT, PT, and TT (Coagulation time cannot be determined, since PPP had already coagulated before being analyzed) compared with control groups, indicating an excellent in vitro hemorrhage control through all the coagulation pathway.

### 2.5 Hemostatic Mechanism of J‐CMH@CaCO_3_/T

**Figure 6:**
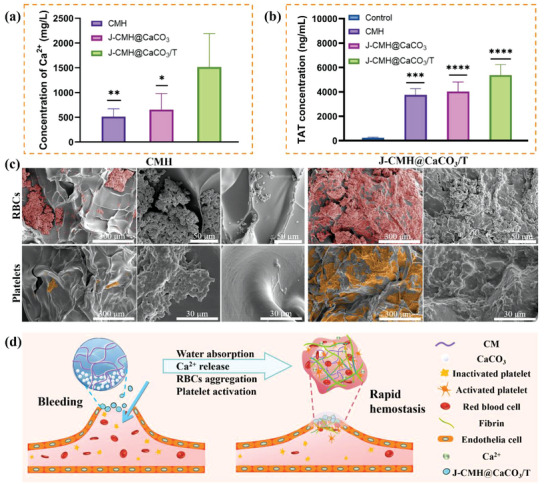
a) Ca^2+^ release concentration of samples. b) TAT expression of blood plasma treated with samples. The blood without any treatment used as control. c) SEM images of RBCs and platelets aggregation on the surface of different samples with different magnifications. d) Schematic illustrations for the hemostasis mechanism of the J‐CMH@CaCO_3_/T.

Under acidic conditions, CaCO_3_ produces Ca^2+^ as well as bubbles. The J‐CMH@CaCO_3_/T was proposed to release Ca^2+^ in bleeding wounds due to its unique structure. Thus, the Ca^2+^ release of the CMH, J‐CMH@CaCO_3_, and J‐CMH@CaCO_3_/T within 2 min was quantitatively presented in **Figure** **6**a. The J‐CMH@CaCO_3_/T had a higher capacity for storing and releasing Ca^2+^. By inducing intrinsic and extrinsic coagulation cascades, the released Ca^2+^ acts as a coagulation cofactor, activating platelets.^[^ ^40^ ^]^ The conversion of prothrombin to thrombin is a crucial step in the coagulation process. As shown in Figure 6b, the thrombin–antithrombin (TAT) concentration of the J‐CMH@CaCO_3_/T was increased dramatically, which directly confirmed the activation of the coagulation system.^[^ ^41^ ^]^ The activation of the coagulation cascade would lead to the generation of thrombin and subsequently to the formation of fibrin networks.^[^ ^42^ ^]^ The hemostatic mechanism of the hydrogel spheres was further investigated by observing the surface aggregation and morphology of erythrocytes and platelets on spheres (Figure 6c). Erythrocytes and platelets were colorized with red and yellow for easier observation, respectively. All the spheres showed large number of blood cells and platelets, which were irregularly aggregated on the surface. Expression of CD41a and CD62P on platelets of the CMH and J‐CMH@CaCO_3_/T was measured by flow cytometry (Figure S3, Supporting Information), showing that the J‐CMH@CaCO_3_/T aggregated more platelets than the CMH in the same period. The spheres based on CM, with a natural positive charge from remaining free amino groups, aggregated negatively charged erythrocytes and platelets effectively through electrostatic interaction.^[^ ^43^ ^]^ The swelling ability of the hydrogel spheres had the advantage of exudate absorption, concentrating blood cells and platelets. The hydrogel spheres could provide a humid environment for wound interface, and avoid secondary injury and prevent loss of body fluids.^[^ ^44^ ^]^ The J‐CMH@CaCO_3_/T showed an increased number of erythrocytes and platelet aggregation. Production of bubbles was accompanied by the release of Ca^2+^ from the J‐CMH@CaCO_3_/T, which could rapidly produce synergistic effects in hemostasis, leading to higher hemostatic efficiency. Also, TXA‐NH_3_^+^ would be deprotonated into TXA‐NH_2_ after neutralizing with CaCO_3_ powder. TXA‐NH_2_ is widely used to treat hemophilia and other coagulopathies as a typical antifibrinolytic drug, avoiding fibrinolysis and preventing the breakdown of blood clots.^[^ ^45^ ^]^ The combination of hemostatic (Ca^2+^) and antifibrinolytic (TXA‐NH_2_) properties is efficient to stop hemorrhage.^[^ ^46^ ^]^ These results indicated that all these factors contributed to the excellent pro‐coagulation performance of the J‐CMH@CaCO_3_/T.

In brief, as shown in Figure 6d, the possible hemostatic mechanism of the J‐CMH@CaCO_3_/T onto the bleeding site was described as follows: the J‐CMH@CaCO_3_/T absorbed the water of serum and wound exudate benefited from the excellent water uptake capacity, then concentrating red blood cells and platelets. Meanwhile, the J‐CMH@CaCO_3_/T penetrated deeply into the wound immediately by self‐propelled property. Then, Ca^2+^ was released to accelerate the blood clotting procedure by taking charge of the hemostatic process, since extracellular Ca^2+^ is required for the final stage of platelet adhesion and clot retraction. Therefore, the J‐CMH@CaCO_3_/T showed excellent hemostatic efficacy to halt hemorrhage.

Biocompatibility is necessary to be assessed for hemostats. Qualified hemostatic materials should not induce significant hemolysis when in contact with bleeding wounds.^[^ ^47^ ^]^ The hemolysis assay was used to evaluate hemocompatibility. After incubating RBCs with the samples at 37 °C for 3 h, the supernatants of all samples obtained by centrifugation showed similar transparency to that of the negative control (Figure S4, Supporting Information). In all groups, the hemolysis ratios were less than 5%, indicating that the hydrogel spheres were at a safe level. The cytocompatibility of the J‐CMH@CaCO_3_/T was investigated using a standard CCK8 method. The cell viability of L929 cells treated with the J‐CMH@CaCO_3_/T for 1 and 5 d at a high concentration of 40 mg mL^−1^ was more than 90%, with no statistically significant difference from that of the positive control (Figure S5, Supporting Information). As further observed by fluorescence cytoskeleton staining (live cells with green signal, dead cells with red signal), the L929 cells showed a normal, regular spindle‐like morphology for the J‐CMH@CaCO_3_/T, revealing that the hydrogel spheres had negligible toxicity^[^ ^48^ ^]^ (Figure S6, Supporting Information).

### 2.6 In Vivo Hemostatic Assay in Rodent Models

**Figure 7:**
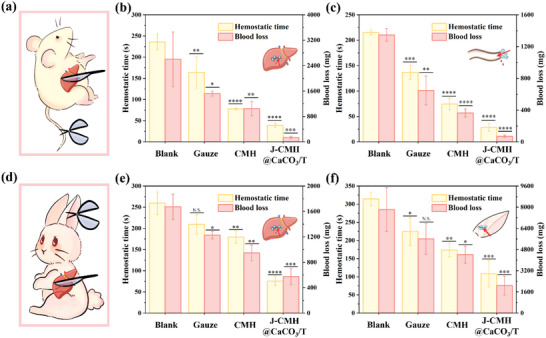
Rats model. a) Schematic illustrations for inducing and stopping bleeding in liver bleeding model and tail amputation. Quantitative results of lost blood from b) bleeding liver and c) tail were treated with different samples. Rabbits model. d) Schematic illustrations for inducing and stopping bleeding in liver and ear artery bleeding model. Quantitative results of lost blood from e) bleeding liver and f) ear were treated with different samples.

Rodent tail and liver bleeding models and rabbits’ ear artery and liver bleeding models were established to evaluate the in vivo hemostatic performance of the J‐CMH@CaCO_3_/T. Different samples were employed to halt the bleeding immediately after inflicting injuries on the tail and the liver of rats (**Figure** **7**a). The hemostasis process in the control and experimental groups of rats bleeding model were photographed in Figure S7a (Supporting Information). The hemostatic properties were further evaluated by quantitative results of blood loss and hemostatic time both in liver injury and tail amputation models. The hemostatic time of the blank group was the longest among all the groups (236 s in the tail bleeding model, 215 s in the liver injury model). For the liver injury model (Figure 7b), the CMH showed a hemostatic time of 74 s, which was shorter than that of the gauze group (136 s). The J‐CMH@CaCO_3_/T stopped the bleeding with the fastest hemostasis time of 28 s and the lowest blood loss of 0.072 g (reduced by 94.6% compared with the blank group), exhibiting superior ability in controlling hemorrhage. For the tail amputation model (Figure 7c), the J‐CMH@CaCO_3_/T showed the shortest hemostatic time of 39 s and the lowest blood loss of 0.138 g (reduced by 95% compared with the blank group). The CMH presented a hemostatic time of 78 s and a blood loss of 1.05 g, but some cases of secondary bleeding after the CMH group removal from the hemostasis wound have occurred in animal experiments. As shown in Figure 7d, ear artery and liver bleeding models of rabbits were also established to evaluate the in vivo hemostatic performance of the J‐CMH@CaCO_3_/T. The hemostasis processes in the rabbits bleeding model were photographed, as shown in Figure S7b (Supporting Information). The J‐CMH@CaCO_3_/T also displayed a distinct hemostatic advantage over medical gauze and the CMH in the rabbit liver bleeding model (Figure 7e) and ear artery bleeding model (Figure 7f). These results demonstrated that the J‐CMH@CaCO_3_/T hydrogel spheres could achieve rapid hemostasis in response to acute injuries. Also, serum calcium concentrations of rabbits were detected after J‐CMH@CaCO_3_/T hydrogel spheres applied on bleeding wounds (Figure S8, Supporting Information). The similar results among the samples were indicated that Ca^2+^ release of the J‐CMH@CaCO_3_/T had no effect on the blood calcium levels of rabbits. The utility of the J‐CMH@CaCO_3_/T showed no toxicity in vivo, which was consistent with in vitro biocompatibility results. It should be noted that TXA‐NH_3_^+^ demonstrated unsatisfactory hemostatic effects both in vitro (Figure S9, Supporting Information) and in vivo studies (Figure S10, Supporting Information), which were attributed to their self‐acidic nature. The acidity of TXA‐NH_3_^+^ might stimulate the wound, prolonging the bleeding time and increasing the amount of bleeding. The unsatisfied hemostatic performance of CaCO_3_/T was also investigated (Figure S11, Supporting Information), related discussions were presented in the Supporting Information. In summary, the J‐CMH@CaCO_3_/T showed excellent hemostatic efficacy both in in vitro and in vivo experiments.

## 3 Conclusions

In conclusion, we put forward a simple protocol to construct the J‐CMH@CaCO_3_/T with self‐propelled property, using an as effective and safe hemostat. The prepared J‐CMH@CaCO_3_ exhibited an asymmetrical structure. The results revealed that the J‐CMH@CaCO_3_/T showed rapid water absorption ability and low cytotoxic impact on L929 cells and RBCs. Also, the J‐CMH@CaCO_3_ exhibited excellent self‐propulsion capacity via a bubble detachment mechanism, accompanying the release of Ca^2+^. In this sense, the J‐CMH@CaCO_3_/T presented an excellent in vitro hemostatic performance. Moreover, in vivo hemostasis indicated that the J‐CMH@CaCO_3_/T stopped bleeding within 39 s in the rodent bleeding models and 109 s in the rabbit bleeding models. Due to these advantages, the J‐CMH@CaCO_3_/T has great potential to halt irregularly shaped, noncompressible hemorrhage. Importantly, this work may provide useful references for the practical application of hemostats, open up a new avenue to design high performance hemostats by gravity.

## 4 Experimental Section

### Materials

Chitosan (CS, deacetylation ≥ 95%, *η* = 100‐200 mPa·s with 10 g CS in 1 L water at 20 °C, from shrimp shells) was purchased from Shanghai Macklin Biochemical Co., Ltd (China). Methacrylic anhydride (MA), calcium carbonate (CaCO_3_), calcium chloride, 2‐hydroxy‐4′‐(2‐hydroxyethoxy)‐2‐methylpropiophenone (photoinitiator I2959), carboxylated chitosan (CCS, *η* = 80 mPa s with 1 wt% CCS in water at 20 °C), tranexamic acid, and sodium hydroxide, were purchased from Shanghai Aladdin Biochemical Technology Co., Ltd (China). All chemical reagents and solvents were reagent grade and used without further purification.

### Synthesis of Chitosan Methacryamide

The preparation steps of chitosan methacryamide (CM) were performed according to a previous method.^[^ ^49^ ^]^ The detailed synthesis and characterization experiments of CM are presented in the Supporting Information.

### Synthesis of TXA‐NH_3_^+^ Powder

The preparation steps of solid organic acids were based on the method reported by Baylis et al.^[^ ^17^ ^]^ Tranexamic acid (TXA‐NH_2_) was dissolved in deionized water (DW), and then HCl was added to adjust the pH of the solution until it reached 4.3. The TXA‐NH_3_^+^ powder was generated after lyophilization.

### Preparation of the Hydrogel Spheres with Shells

The CM was dissolved in water at a concentration of 1.5 wt%. Subsequently, 0.75 g CaCO_3_ powder was added into a mixed suspension of calcium chloride (6 wt%) and I2959 (0.25 wt%), and the mixture was magnetically stirred at room temperature as a spinning solution. The coagulation bath contained 1 wt% CCS in a water‐ethanol system (w/w = 3:7). The as‐prepared spinning solution (in a 5 mL syringe) was dropped in the coagulation bath under a high voltage (12 kV) with a feeding rate of 1.2 mm min^−1^. Then, hydrogel spheres with shells formed by partial crosslinking of CCS and Ca^2+^ were obtained and then stored in DW for further use.

### Preparation of the Hydrogel Spheres

To prepare J‐CMH@CaCO_3_, the hydrogel spheres with shells were placed in a glass petri dish until CaCO_3_ was unevenly distributed in the spheres by gravity settlement. The polymerization was initiated by UV irradiation for 10 min. After that, the cross‐linked hydrogel spheres with shells were immersed in a 0.01 mol L^−1^ NaOH solution with rapid stirring to dissolve the CCS/Ca^2+^ shell. Then, J‐CMH@CaCO_3_ were obtained after being frozen at −40 °C and lyophilized for 24 h. The formulation of the hydrogel spheres was listed in Table S1 (Supporting Information).

### Preparation of Self‐Propelled Hemostats

To endow the hydrogel spheres with self‐propelling property, the TXA‐NH_3_^+^ powder was blended with the J‐CMH@CaCO_3_ by using an equal molar ratio of CaCO_3_, labeled as the J‐CMH@CaCO_3_/T. Individual hydrogel spheres of J‐CMH@CaCO_3_ had the same CaCO_3_ powder content, because the spinning solution was slightly viscous, which led to a good suspension and dispersion of CaCO_3_ powder dispersed in the spinning solution during the dropping process. The content of CaCO_3_ was quantitatively evaluated by the weight of hydrogel spheres.

### Morphology of the Hydrogel Spheres

The morphology of the hydrogel spheres was explored under scanning electron microscopy (SEM), digital photos, and optical microscope.

### Water Absorption Tests

The water adsorption ratio of the hydrogel spheres was confirmed by weighing and measuring the spheres after immersing in DW, EDTA (K2)‐anticoagulated whole blood (WB), and normal saline (NS, 0.9% NaCl) at room temperature. The water contact angles (WCAs) of the hydrogel spheres were determined. The detailed procedures are shown in the Supporting Information.

### Self‐Propelled Behavior Tests

The self‐propelled behavior of the hydrogel spheres in DW was observed by the camera and inverted optical microscope. The maximum self‐propelling distance was measured in individual glass tubes containing DW or WB to assess the self‐propulsion property. The glass tubes were fixed at various angles in the horizontal direction.

### In Vitro Blood‐Related Component Preparation

New Zealand rabbits (2.5 ± 0.2 kg, 3 months, male) were used for blood‐related experiments. Red blood cells (RBCs), and platelet‐poor plasma (PPP) of rabbits were prepared for further tests. Coagulation pathway analysis and enzyme‐linked immunosorbent assays (ELISA) tests were carried out using human blood. The whole blood was donated by a 26 years old male. The detailed steps are given in the Supporting Information.

### In Vitro Biocompatibility Tests

To evaluate the hemocompatibility, red blood cells prepared from rabbits’ whole blood were explored to contact directly with the hydrogel spheres. The cytotoxicity of the hydrogel spheres on L929 fibroblasts was evaluated using two methods, including CCK‐8 assay and Live/Dead Viability Kit assay. The detailed procedures are given in the Supporting Information.

### In Vitro Hemostatic Property Tests

The hemostatic mechanism of the hydrogel spheres was evaluated via BCI, platelets and RBCs aggregation assays, commercial ELISA, and coagulation pathway analysis in vitro. Gauze, CMH, and CMH@CaCO_3_ were used as control groups in these tests. The detailed procedures are given in the Supporting Information.

### In Vivo Hemostatic Process

Sprague‐Dawley rats (SD rats, 225 ± 25 g, 6 weeks, female) and New Zealand white rabbits (2.5 ± 0.2 kg, 3 months, male) were both used for in vivo experiments (rodent tail amputation, liver‐bleeding model of rats and rabbits, rabbit‐ear artery bleeding model) according to Table S2 (Supporting Information). Samples were applied to the wounds to investigate the hemostasis time and blood loss for all groups. Lost blood was calculated by weighing the filter paper before and after absorbing blood, and the bleeding duration was recorded simultaneously. The detailed procedures are given in the Supporting Information.

### Animal Welfare

All procedures involving the use of animals in this study were performed in strict accordance with the Guide for the Care and Use of Laboratory Animals. All animal experiments were prospectively approved by the ethics committee (No. 2021911A) of West China Hospital, Sichuan University. During the experiment, the animals had free access to water and food.

### Statistical Analysis

All the acquired data were presented as mean ± standard deviation (SD) from at least three parallel experiments per group. Independent t test and two‐way ANOVA were used to evaluate the differences among the groups. The statistical significance was considered significant and marked as *p < 0.05, **p < 0.01, ***p < 0.001, ****p < 0.0001, and N.S. for no significant differences.

## Conflict of Interest

The authors declare no conflict of interest.

## Author Contributions

Q.Y. performed investigation, formal analysis, visualization, and wrote the original draft. B.S. performed methodology and conceptualization. W.Z. performed conceptualization, project administration, and supervision. C.Z. performed supervision.

## Supporting information

Supporting Information

Supplemental Movie 1

Supplemental Movie 2

Supplemental Movie 3

## Data Availability

The data that support the findings of this study are available from the corresponding author upon reasonable request.

## References

[1] Y. Wang , D. Xiao , Y. Zhong , Y. Liu , L. Zhang , Z. Chen , X. Sui , B. Wang , X. Feng , H. Xu , Z. Mao , Int. J. Biol. Macromol. 2020, 160, 18. 32428591 10.1016/j.ijbiomac.2020.05.099

[2] F. Maisano , H. K. Kjaergård , R. Bauernschmitt , A. Pavie , G. Rábago , M. Laskar , J. P. Marstein , V. Falk , Eur. J. Cardio‐thoracic Surg. 2009, 36, 708. 10.1016/j.ejcts.2009.04.057 19595605

[3] E. M. Singletary , D. A. Zideman , J. C. Bendall , D. A. Berry , V. Borra , J. N. Carlson , P. Cassan , W.‐T. Chang , N. P. Charlton , T. Djärv , M. J. Douma , J. L. Epstein , N. A. Hood , D. S. Markenson , D. Meyran , A. Orkin , T. Sakamoto , J. M. Swain , J. A. Woodin , E. De Buck , N. De Brier , D. O , C. Picard , C. Goolsby , E. Oliver , B. Klaassen , K. Poole , T. Aves , S. Lin , A. J. Handley , et al., Resuscitation 2020, 156, A240. 33098920 10.1016/j.resuscitation.2020.09.016

[4] H. Khoshmohabat , S. Paydar , A. Makarem , M. Y. Karami , N. Dastgheib , S. A. H. Zahraei , R. Rezaei , G. S. Mahmoudi Nezhad , Open Access Emerg. Med. 2019, 11, 171. 31534375 10.2147/OAEM.S205006 PMC6682168

[5] L. Yu , X. Shang , H. Chen , L. Xiao , Y. Zhu , J. Fan , Nat. Commun. 2019, 10, 1932. 31036816 10.1038/s41467-019-09849-9 PMC6488602

[6] Y. Hao , W. Zhao , L. Zhang , X. Zeng , Z. Sun , D. Zhang , P. Shen , Z. Li , Y. Han , P. Li , Q. Zhou , Mater. Des. 2020, 193, 108863.

[7] J. B. Holcomb , J. M. McClain , A. E. Pusateri , D. Beall , J. M. Macaitis , R. A. Harris , M. J. MacPhee , J. R. Hess , J. Trauma: Inj., Infect., Crit. Care 2000, 49, 246. 10.1097/00005373-200008000-00010 10963535

[8] M. B. Dowling , W. Smith , P. Balogh , M. J. Duggan , I. C. MacIntire , E. Harris , T. Mesar , S. R. Raghavan , D. R. King , J. Surg. Res. 2015, 193, 316. 25016441 10.1016/j.jss.2014.06.019

[9] G. Z. Xu , L. Cheng , Q. T. Zhang , Y. L. Sun , C. L. Chen , H. Xu , Y. M. Chai , M. D. Lang , J. Biomater. Appl. 2016, 31, 721. 27485953 10.1177/0885328216661557

[10] X. M. Zhang , L. Jiang , X. Li , L. W. Zheng , R. Y. Dang , X. Liu , X. P. Wang , L. L. Chen , Y. S. Zhang , J. X. Zhang , D. Q. Yang , Small 2022, 18, 2101699. 10.1002/smll.202101699 34817129

[11] X. Zhao , Y. P. Liang , B. L. Guo , Z. H. Yin , D. Zhu , Y. Han , Chem. Eng. J. 2021, 403, 126329.

[12] M. B. Dowling , I. C. MacIntire , J. C. White , M. Narayan , M. J. Duggan , D. R. King , S. R. Raghavan , ACS Biomater. Sci. Eng. 2015, 1, 440. 33445247 10.1021/acsbiomaterials.5b00067

[13] C. Hong , B. D. Olsen , P. T. Hammond , Biomaterials 2022, 283, 121432. 35245732 10.1016/j.biomaterials.2022.121432

[14] H. B. Li , F. Cheng , X. J. Wei , X. T. Yi , S. Z. Tang , Z. Y. Wang , Y. S. Zhang , J. M. He , Y. D. Huang , Mater. Sci. Eng. C 2021, 118, 111324. 10.1016/j.msec.2020.111324 33254961

[15] S. C. Lu , X. H. Zhang , Z. W. Tang , H. Xiao , M. Zhang , K. Liu , L. H. Chen , L. L. Huang , Y. H. Ni , H. Wu , Chem. Eng. J. 2021, 417, 129329.

[16] Y. Wang , P. Zhou , D. Xiao , Y. Liu , Y. Zhong , B. Wang , L. Zhang , Z. Chen , X. Sui , X. Feng , H. Xu , Z. Mao , Cellulose 2020, 27, 10139.

[17] J. R. Baylis , J. H. Yeon , M. H. Thomson , A. Kazerooni , X. Wang , A. E. St John , E. B. Lim , D. Chien , A. Lee , J. Q. Zhang , J. M. Piret , L. S. Machan , T. F. Burke , N. J. White , C. J. Kastrup , Sci. Adv. 2015, 1, e1500379. 26601282 10.1126/sciadv.1500379 PMC4646796

[18] Q. Li , E. Hu , K. Yu , R. Xie , F. Lu , B. Lu , R. Bao , T. Zhao , F. Dai , G. Lan , Adv. Funct. Mater. 2020, 30, 2004153.

[19] J. Hu , S. X. Zhou , Y. Y. Sun , X. S. Fang , L. M. Wu , Chem. Soc. Rev. 2012, 41, 4356. 22531991 10.1039/c2cs35032g

[20] Y. P. Duan , X. Zhao , M. M. Sun , H. Hao , Ind. Eng. Chem. Res. 2021, 60, 1071.

[21] X. Wang , L. Baraban , V. R. Misko , F. Nori , T. Huang , G. Cuniberti , J. Fassbender , D. Makarov , Small 2018, 14, 1803613. 10.1002/smll.201802537 30238700

[22] Z. H. Nie , W. Li , M. Seo , S. Q. Xu , E. Kumacheva , J. Am. Chem. Soc. 2006, 128, 9408. 16848476 10.1021/ja060882n

[23] S. Lone , I. W. Cheong , RSC Adv. 2014, 4, 13322.

[24] L. Feng , W. Shi , Q. Chen , H. Cheng , J. Bao , C. Jiang , W. Zhao , C. Zhao , Adv. Healthcare Mater. 2021, 10, 2100784. 10.1002/adhm.202100784 34050632

[25] X. Huang , D. Zhou , Y. Wang , C. He , W. Zhao , S. Sun , C. Zhao , Chem. Eng. J. 2019, 359, 1360.

[26] W. T. Kang , B. Bi , R. X. Zhuo , X. L. Jiang , Carbohydr. Polym. 2017, 160, 18. 28115092 10.1016/j.carbpol.2016.12.032

[27] J. Y. Liu , Y. Xiao , X. Y. Wang , L. X. Huang , Y. Chen , C. Y. Bao , Int. J. Biol. Macromol. 2019, 122, 19. 30287380 10.1016/j.ijbiomac.2018.09.202

[28] S. Kim , Y. Q. Kang , A. E. Mercado‐Pagan , W. J. Maloney , Y. Z. Yang , J. Biomed. Mater. Res., Part B 2014, 102, 1393. 10.1002/jbm.b.33118 24500890

[29] R. Lavanya , T. Gomathi , K. Vijayalakshmi , M. Saranya , P. N. Sudha , S. Anil , Int. J. Biol. Macromol. 2017, 104, 1495. 28472686 10.1016/j.ijbiomac.2017.04.116

[30] M. Rasoulzadeh , H. Namazi , Carbohydr. Polym. 2017, 168, 320. 28457456 10.1016/j.carbpol.2017.03.014

[31] K. H. Lee , H. Y. Kim , H. J. Bang , Y. H. Jung , S. G. Lee , Polymer 2003, 44, 4029.

[32] J. S. Choi , Y. Kim , J. Kang , S. Y. Jeong , H. S. Yoo , AAPS PharmSciTech 2013, 14, 794. 23636817 10.1208/s12249-013-9965-x PMC3665992

[33] L. Gao , J. Chen , W. Feng , Q. Song , J. Huo , L. Yu , N. Liu , T. Wang , P. Li , W. Huang , Biomater. Sci. 2020, 8, 6930. 32964904 10.1039/d0bm00800a

[34] A. Bortolin , F. A. Aouada , E. Longo , L. Mattoso , Polímeros 2011, 22, 311.

[35] M. Mathesh , J. W. Sun , F. van der Sandt , D. A. Wilson , Nanoscale 2020, 12, 22495. 33169767 10.1039/d0nr04415f

[36] Y. Huang , X. Zhao , Z. Zhang , Y. Liang , Z. Yin , B. Chen , L. Bai , Y. Han , B. Guo , Chem. Mater. 2020, 32, 6595.

[37] G. Q. Lan , Q. Li , F. Lu , K. Yu , B. T. Lu , R. Bao , F. Y. Dai , Cellulose 2020, 27, 385.

[38] Q. Li , E. Hu , K. Yu , M. Lu , R. Xie , F. Lu , B. Lu , R. Bao , G. Lan , Bioact. Mater. 2021, 6, 4625. 34095621 10.1016/j.bioactmat.2021.05.006 PMC8141897

[39] C. d'Audigier , C. Delassasseigne , A. Robert , V. Eschwege , Int. J. Lab. Hematol. 2016, 38, 50. 10.1111/ijlh.12434 26406495

[40] J. J. Zhu , Y. B. Sun , W. Z. Sun , Z. Y. Meng , Q. L. Shi , X. X. Zhu , H. Gan , R. L. Gu , Z. N. Wu , G. F. Dou , Int. J. Biol. Macromol. 2019, 134, 435. 31100389 10.1016/j.ijbiomac.2019.05.086

[41] M. Weber , F. Umrath , H. Steinle , L.‐F. Schmitt , L.‐T. Yu , C. Schlensak , H‐P. Wendel , S. Reinert , D. Alexander , M. Avci‐Adali , Int. J. Mol. Sci. 2021, 22, 9942. 34576103 10.3390/ijms22189942 PMC8467579

[42] K. F. Lei , K.‐H. Chen , P.‐H. Tsui , N.‐M. Tsang , PLoS One 2013, 8, e76243. 24116099 10.1371/journal.pone.0076243 PMC3792124

[43] H. Hattori , M. Ishihara , Biomed. Mater. 2015, 10, 015014. 25611127 10.1088/1748-6041/10/1/015014

[44] J. Xiang , L. Shen , Y. Hong , Eur. Polym. J. 2020, 130, 109609.

[45] L. Tengborn , M. Blomback , E. Berntorp , Thromb. Res. 2015, 135, 231. 25559460 10.1016/j.thromres.2014.11.012

[46] I. Denry , J.‐M. Nedelec , J. A. Holloway , J. Biomed. Mater. Res., Part B 2022, 110, 422. 10.1002/jbm.b.34918 PMC8678343 34288380

[47] X. J. Sun , J. Li , K. Shao , C. Su , S. C. Bi , Y. Z. Mu , K. C. Zhang , Z. Cao , X. Y. Wang , X. G. Chen , C. Feng , Int. J. Biol. Macromol. 2021, 182, 2097. 34081956 10.1016/j.ijbiomac.2021.05.123

[48] K. C. Zhang , J. Li , Y. N. Wang , Y. Z. Mu , X. J. Sun , C. Su , Y. Dong , J. H. Pang , L. Huang , X. G. Chen , C. Feng , Carbohydr. Polym. 2020, 236, 9. 10.1016/j.carbpol.2020.116051 32172865

[49] L. D. Zhu , K. M. Bratlie , Biochem. Eng. J. 2018, 132, 38.

