# Supplementary material for: Janus Self‐Propelled Chitosan‐Based Hydrogel Spheres for Rapid Bleeding Control
Source: Adv Sci (Weinh). 2022 Dec 25;10(5):2205989. doi: 10.1002/advs.202205989 (PMC9929117; doi:10.1002/advs.202205989)
Supplement: Supplementary file 1 — Supporting Information [file ADVS-10-2205989-s002.pdf]

ADVANCED SCIENCE
Open Access

Supporting Information

for *Adv. Sci.*, DOI 10.1002/advs.202205989

Janus Self-Propelled Chitosan-Based Hydrogel Spheres for Rapid Bleeding Control

Qiao Yu, Baihai Su, Weifeng Zhao* and Changsheng Zhao*

Supporting Information

Self-propelling Janus Chitosan-Based Hydrogel Beads by Gravity Settling for Rapid Bleeding Control

Qiao Yu[a, d], Baihai Su[b, d*], Weifeng Zhao[c,d*], and Changsheng Zhao[c,d]

[a] *Institute for Disaster Management and Reconstruction, Sichuan University, Chengdu 610207, China.*

[b] *Department of Nephrology, West China Hospital, Sichuan University, Chengdu 610041, China.*

[c] *College of Polymer Science and Engineering, State Key Laboratory of Polymer Materials Engineering, Sichuan University, Chengdu 610054, China.*

[d] *Med-X Center for Materials, Sichuan University, Chengdu, China.*

1. Experimental Section

Synthesis of chitosan methacryamide (CM)

Chitosan was firstly dissolved in 2 wt.% acetic acid under constant stirring to obtain a 3 wt.% chitosan dispersion. Methacrylic anhydride (MA) was added dropwise into the chitosan dispersion (The molar ratio of MA to amino groups on the chitosan backbone was 1:2). The mixture was homogenized and reacted by stirring for 4 h at 50 °C. Afterward, the milky solution was purified through dialysis (MWCO: 7000 Da) against deionized water (DW) for 3 days. The CM was obtained and collected as a cotton-like white solid by lyophilization.

Preparation of the hydrogel spheres

Table S1. The formulation of hydrogel spheres

Name	CM, I2959, $CaCl_2$	$CaCO_3$	Gravity settlement
J-CMH@$CaCO_3$	√	√	√
CMH@$CaCO_3$	√	√	/
CMH	√	/	/

Characterization of CM

The chemical structure of CM was characterized by Fourier transform infrared spectroscopy (FTIR) and thermal gravimetric analysis (TGA). FTIR analysis was investigated by FTIR spectrometer (FTIR, Nicolet iS50, USA) recorded from 400 to 4000 cm^{-1}, and the samples were completely dried and mixed with KBr pellets at room temperature. TGA was carried out on a thermos gravimetric analyzer (METTLLE TOLEDO, TGA/DSC 3+, Switzerland) at 40 to 800 °C with a heating rate of 10 °C/min under nitrogen flow.

Morphology of the hydrogel spheres

Scanning electron microscopy (SEM) was used to observe the surface morphology, by using a scanning electron microscope (JMS-7500F SEM JEOL, Japan) after samples were coated with gold.

Water absorption tests

The surface water contact angles (WCAs) were investigated by a video capture system with 2 μL of a water droplet (SL200KS, Surface-science, China).

The water adsorption ratio of hydrogel spheres was confirmed by weighing and measuring in DW, normal saline (NS, 0.9% NaCl), and EDTA (K2)-anticoagulated whole blood of rabbits (WB) at room temperature. Dried samples were immersed in different solutions, and then the samples were removed from the solutions by centrifugation and wiped gently with filter paper. The weights of dry and wet samples were weighted and recorded as W_{dry} and W_{wet}, respectively. The water absorption ratio was calculated from eq. (1).

$$\text{Water absorption ratio} = \frac{W_{wet}-W_{dry}}{W_{dry}} \times 100\% \qquad \text{eq. (1)}$$

In vitro blood-related component preparation

Citrate-anticoagulated whole blood (CWB) was prepared by extracting whole blood from the auricular veins of a healthy New Zealand White Rabbit for 3.8% sodium citrate. Platelet-poor plasma (PPP) was obtained as the supernatant by centrifugation of CWB under 4000 rpm for 15 min. The residual part of the blood could be used to extract red blood cells (RBCs), which were obtained by centrifugation of CWB under 2000 rpm for 15 min and purified five times with NS.

In vitro hemostatic property tests

Blood-clotting index

Samples were cast in a culture dish and covered at the bottom of the well. 200 μL CWB with 20 μL $CaCl_2$ solution (0.1M) was dropped on the surface of the sample. After initiating blood coagulation at 37 °C for 5 min, 25ml DW was added to the culture dish. The supernatant was collected after the incubation for 10 min, and the absorbance was measured at 540 nm using a UV spectrophotometer (UV-1750, Shimadzu Co., Ltd, Japan). The blood treated with $CaCl_2$ was recorded as $A_{reference}$, samples were recorded as A_{sample}, and BCI was calculated by eq. (2).

$$\text{BCI} = \frac{A_{sample}-A_{DI\,water}}{A_{reference}-A_{DI\,water}} \times 100\% \qquad \text{eq. (2)}$$

Whole blood-clotting time test (WBCT)

10 mg of samples were placed in a 24-well tissue culture plate. 100 μL of CWB was added into each well to fully immerse the samples and incubated at 37 °C for a predefined time. The uncoagulated solution was removed, and the well was washed three times with PBS. Blood clotting was observed every 15 s, and clotting time was recorded. The CWB treated with $CaCl_2$ served as a control.

Hemostatic mechanism

Adhesive state of red blood cells and platelet

The adhesion state of blood cells and platelets also indicates the blood coagulation effect of hemostatic materials by representing platelet activation[1]. More blood cell adhesion indicates better hemorrhage control. Samples were added to 24-well tissue culture plates after rewarming at 37 °C. Following that, 200 μL of RBCs (5%, diluted with NS) or PRP were added dropwise into each well and then incubated at 37 °C for 1 h. All the samples were washed with PBS solution three times to remove the no-adhered erythrocytes and platelets, and then fixed with 2.5% glutaraldehyde for 2 h. The samples were observed using SEM after being gradually dehydrated by 30%, 50%, 70%, 80%, 90%, 95%, and 100% ethanol with a time interval of 15 min.

Coagulation pathway analysis

Activated partial thromboplastin time (APTT), prothrombin time (PT), and thrombin time (TT) were used to analyze the coagulation pathway. Each sample was mixed with 200 μL of PPP and warmed at 37 °C in a water bath for 15 min. Subsequently, PPP was extracted and added to the test tube. APTT, PT, and TT values were evaluated by an automatic coagulation analyzer (CA500, Sysmex Corporation, Japan). The upper detect limits (APTT > 600 s and TT > 180 s) of the automated blood coagulation analyzer are defined as non-coagulation.

Commercial enzyme-linked immunosorbent assay (ELISA)

ELISA was carried out to evaluate contact activation (TAT, Assaypro LLC, USA) of samples. The samples were added to 24-well plates, and 250 μL of human whole blood was introduced after rewarming for 1 h. After being incubated at 37 °C for 1 h,

the whole blood was centrifuged for 10 min at 2500 g to obtain plasma. The obtained plasma was diluted to the required concentration and added to antibody-coated wells (provided by kit). The detections were performed according to the respective instruction manuals. Pristine whole blood was used as a blank control.

Flow cytometry analysis

Monoclonal antibodies against CD41a (GP IIb) and CD62P (GMP-140) were obtained from Becton, Dickinson and Company (USA) and utilized in flow cytometry analysis. CWB was immediately mixed with the J-CMH@$CaCO_3$/T for 2 min at room temperature and fixed with 1% paraformaldehyde for 2 h at 4 °C. Platelet-rich plasma (PRP) was obtained as the supernatant by centrifugation of CWB under 1000 rpm for 5 min, and followed by washing with buffer solution. PRP was labeled with saturating concentrations of monoclinal antibodies at room temperature in the dark for further flow cytometry analysis. The CWB treated with the CMH was used as a control.

In the above experiments, fresh blood without any anticoagulant was donated by an adult, approved by West China Hospital, Sichuan University (K2016027), and performed in compliance with relevant laws and national guidelines (GB/T 16886.4-2003/ISO 10993-4:2002, General Administration of Quality Supervision, Inspection and Quarantine of the People's Republic of China, Standardization Administration of the People's Republic of China). Informed consent was obtained for any experimentation with human subjects.

The Ca^{2+} release

200 mg of samples were soaked in 25 mL of PBS buffer solution under gently shaking at 37 °C for 120 s, and 2 mL of the supernatant solution was taken to measure the Ca^{2+} concentration by an atomic absorption spectrometer (AAS, SpectrAA 220FS/220Z, USA).

In vitro biocompatibility tests

Hemolysis ratio

Samples and RBCs suspension (0.2 mL of 10% RBCs and 0.8 mL PBS) were added into a 2-mL tube. 0.2 mL of 10% RBCs suspension treated with 0.8 mL PBS and DW

served as negative control and positive control, respectively. After being incubated at 37 °C for 3 h, all the samples were centrifuged at 8000 rpm for 3 min to obtain supernatants. The absorbance of the released hemoglobin in supernatants was measured at 540 nm. The hemolysis ratio was calculated by eq. (3).

$$\text{Hemolysis ratio} = \frac{OD_{sample} - OD_{negative}}{OD_{positive} - OD_{negative}} \times 100\% \qquad \text{eq. (3)}$$

Cytotoxicity

The cytotoxicity of the samples on L929 fibroblast cells was evaluated using the leaching method. Samples were sterilized by immersing in 75% alcohol. Then, leachate was extracted after that the samples were immersed in Dulbecco's modified eagle medium (DMEM, Thermo Fisher Scientific, USA) with 10% fetal bovine serum and 1% penicillin-streptomycin in an incubator for 24 h under 37 °C. L929 cells, and the leaching solutions were introduced into a 96-well plate at a density of 10,000 cells/well. After being cultured for 5 days in an incubator (5% CO_2), the cell viability was evaluated by LIVE/DEAD® Viability/Cytotoxicity Kit assays (CA1630, Solarbio Science & Technology Co., Ltd, China). Afterward, the cells were stained with Calcein AM and PI at room temperature and recorded by a fluorescent inverted microscope (LEICA DMi8, Germany). The CCK-8 assays were also used to investigate cell viability. 100 μL of CCK-8 (final dilution=1:10) was added to each well. After incubating at 37 °C for 2 h, the absorbance of the supernatant was read at 450 nm using a UV spectrophotometer. Normal DMEM medium with/without cells were used as control groups, recorded as OD_{blank} and $OD_{nocells}$. Relative cell viability was calculated by eq. (4).

$$\text{Relative cell viability} = \frac{OD_{sample} - OD_{nocells}}{OD_{Blank} - OD_{nocells}} \times 100\% \qquad \text{eq. (4)}$$

In vivo hemostatic process

In vivo tests are believed to be more convenient for observing the overall effects of the experiments on a living subject. We combined the designs of *in vivo* hemostatic assays of different papers and then chose four bleeding models for *in vivo* hemostatic performance experiments and different models to represent different bleeding

situations.

Table S2. Comparison among *in vivo tests* of hemostats

No.	Year	Rats		Rabbits		Other bleeding models	Reference
		Liver	Tail	Ear artery	Liver		
1	2022	√	-	-	-	-	Zou *et al.*[2]
2	2022	√	-	-	-	-	Yang *et al.*[3]
3	2022	-	-	-	√	-	Zou *et al.*[4]
4	2022	√	-	-	-	Rats-heart	Zhang *et al.*[5]
5	2021	√	-	-	√	Swine-subclavian artery and vein	Zhao *et al.*[6]
6	2021	√	√	√	√	-	Cheng *et al.*[7] Our team
7	/	√	√	√	√	-	Our work

Rodents, including rats, remain the model of choice for studies of physiology, behavior, and complex human disease due to the advantages of cost, reproduction, and feeding[8]. The rat-amputation model represents rapid bleeding, and the rat-liver hemorrhage model represents chronic bleeding. Sprague-Dawley rats (SD rats, 225 ± 25 g, 6 weeks, female) as rodent bleeding models, obtained from West China Hospital of Sichuan University, were used for *in vivo* experiments. Rats were anesthetized intramuscularly. The abdominal region of the rats was shaved and sterilized with saline. For rat-liver injury, their livers were exposed through an abdominal incision at a cut of 1.0 cm × 0.5 cm (length and depth). For rat-tail amputation, rats were amputated tail at the position of 50% length (10 cm) by a surgical cutting tool. After 15 s of bleeding to make sure of normal blood loss, the samples were placed over the incision successively. Blood and serous fluid were carefully collected with pre-weighed filter papers to investigate the blood loss[9], the bleeding duration was recorded simultaneously.

Rabbits also have advantages as animal models for medical procedures and surgery due to the low cost of acquisition and docility behavior. Compared with rodent bleeding models, rabbit bleeding models have a massive hemorrhage. New Zealand white rabbits (2.5 ± 0.2 kg, 3 months, male), obtained from West China Hospital of Sichuan University, and were used for *in vivo* experiments. The rabbits were anesthetized by intravenous injection into the ear, and then fixed onto the operating table. The abdominal region was shaved and sterilized with saline. For rabbit-ear artery bleeding, the rabbit's ear artery was cut off by a surgical cutting tool. For rabbit-liver injury, their livers were exposed through an abdominal incision at a cut of 2.0 cm × 0.5 cm (length and depth). After 15 s of bleeding to make sure of normal blood loss, the samples were placed over the incision successively. Blood and serous fluid were carefully collected with pre-weighed filter papers to investigate the blood loss, the bleeding duration was recorded simultaneously. After being observed for 1 h, the blood in the rabbits was collected for a serum calcium test, then the rabbits were finally euthanized with an overdose of sodium pentobarbital.

We believed that 4 bleeding models to perform *in vivo* hemostatic experiments could confirm the suitability of the hydrogel spheres to deal with different hemostatic situations and different types of wounds.

Hemostatic process of $CaCO_3$/T

For *in vitro* blood-clotting experiment, 200 μL of CWB treated with $CaCl_2$ was added to fully immerse 10 mg of the J-CMH@$CaCO_3$/T and $CaCO_3$/T. At determined time intervals, 15 mL of deionized water was added to wash the RBCs that were not trapped by blood clots. The supernatant was collected after incubation for 10 min and the absorbance at 540 nm was measured using a UV spectrophotometer. For *in vivo* hemostasis experiment, the hemostatic effect was observed by applying the $CaCO_3$/T onto rabbit-ear artery bleeding model.

2. Results and discussion

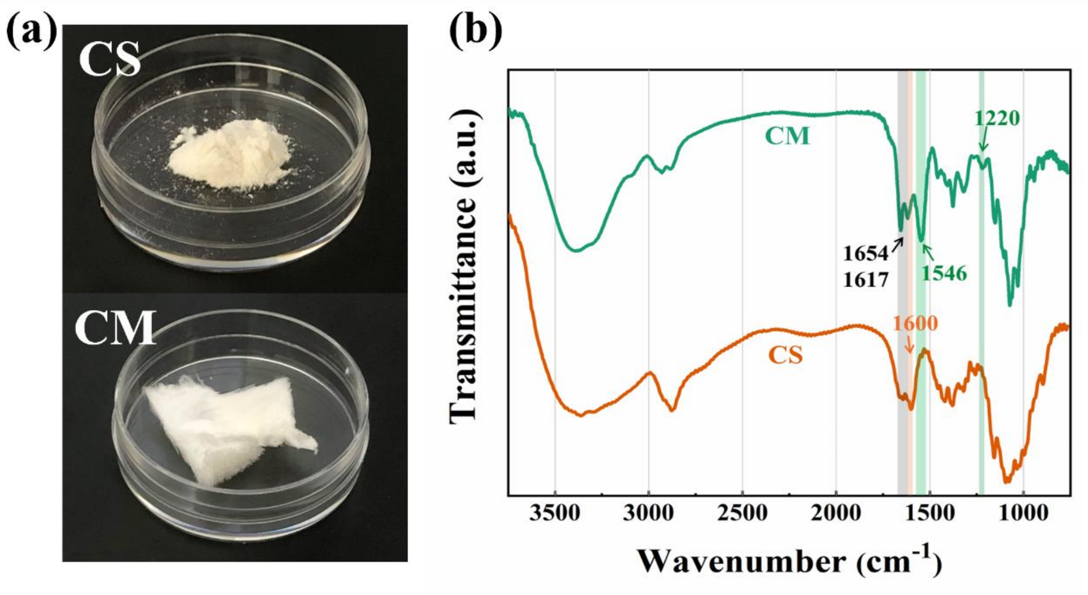

Figure S1. Digital photos (a), and FTIR (b) of CS and CM.

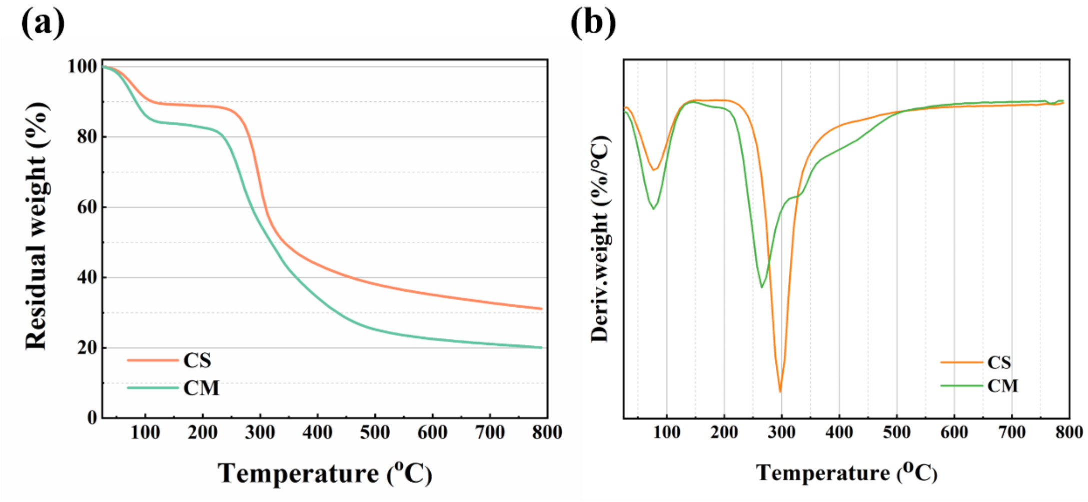

Figure S2. TGA (a), and differential thermogravimetry (DTG, b) curves of CS and CM.

Table S3. APTT, PT, and TT of different samples

Name	APTT (s)	PT (s)	TT (s)
**Blood**	40.56±0.43	11.9±0.2	27.1±3.1
**Gauze**	45.0±1.76	11.67±0.43	28.46±2.73
**CMH**	-	-	-
**J-CMH@$CaCO_3$/T**	-	-	-

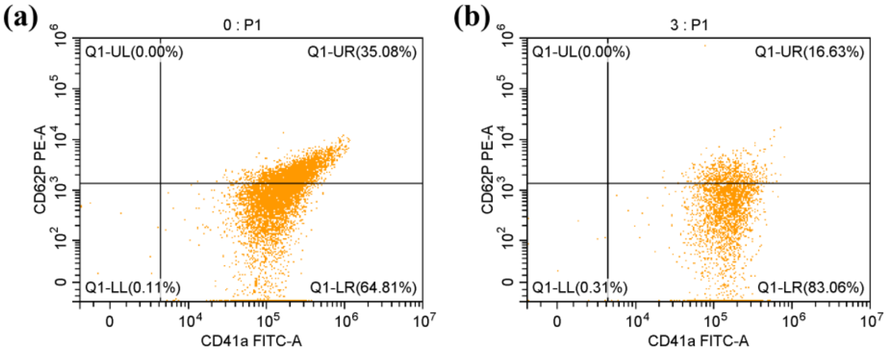

Figure S3. The expression of CD41a and CD62P on platelets of the CMH (a) and the J-CMH@$CaCO_3$/T (b) was measured by flow cytometry.

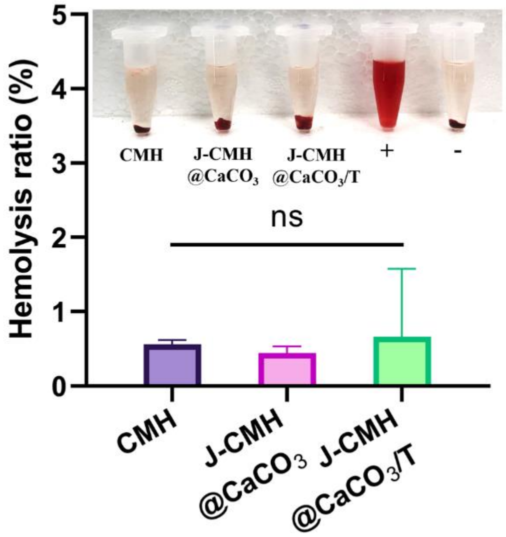

Figure S4. Hemolysis ratios, with an inserted image of samples treated with RBCs. Positive control: RBCs treated with DI (+), negative control: RBCs treated with NS (-)

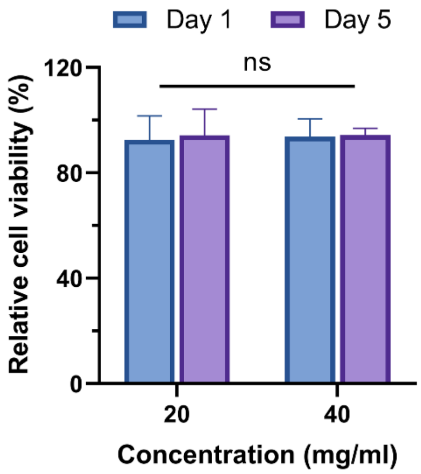

Figure S5. Relative cell viability of L929 cells cultured with J-CMH@$CaCO_3$/T for 1 and 5 days.

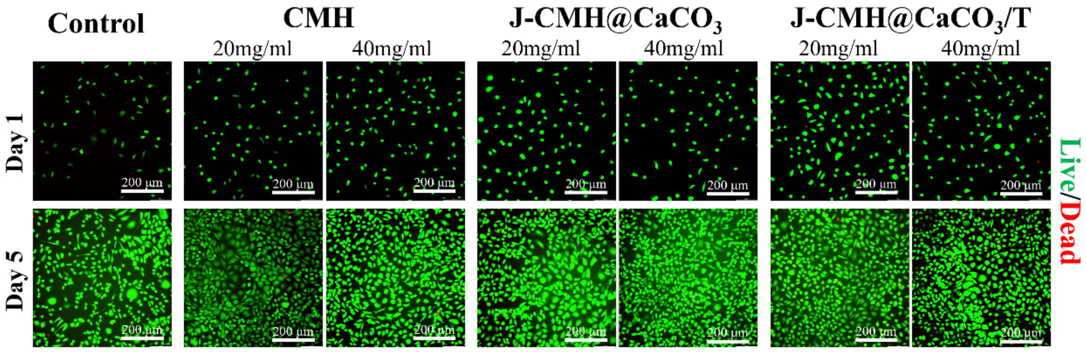

Figure S6. Live/Dead staining of L929 cells after incubation with different concentrations of samples. Negative control: cells cultured in a medium without hydrogel spheres.

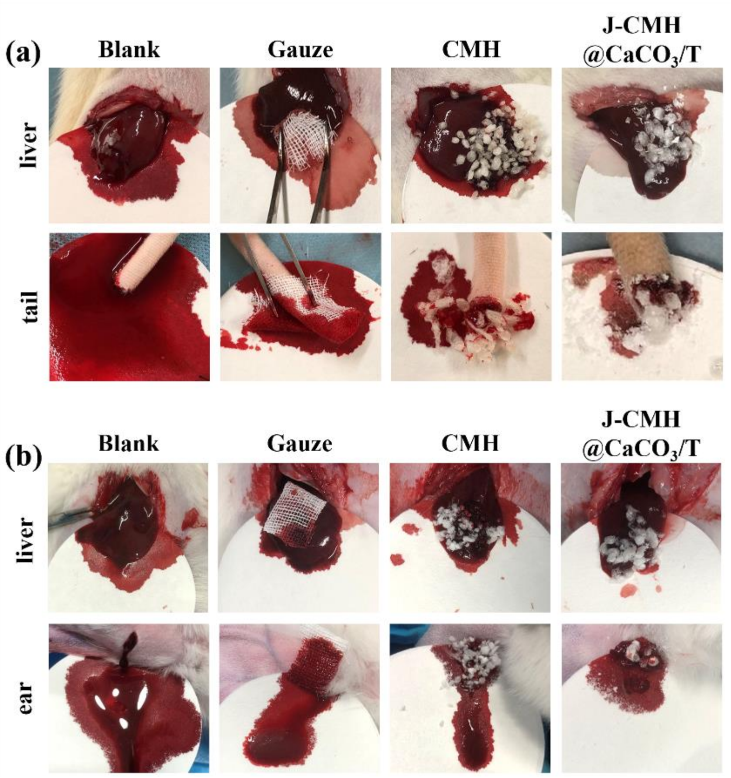

Figure S7. (a) Images of hemostasis in liver bleeding model and tail amputation of rats after depositing different samples. (b) Images of hemostasis in liver and ear artery bleeding model of rabbits after depositing different samples.

(a)

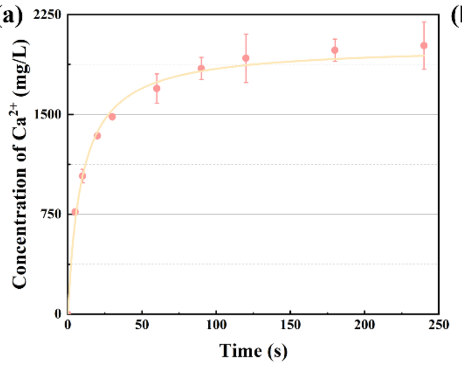

(b)

Sample	Serum Calcium Concentration (mmol/L)
Blank	3.775±0.062
Gauze	3.655±0.044
CMH	3.585±0.055
J-CMH@$CaCO_3$/T	3.483±0.065

Figure S8. (a) Ca^{2+} release capability of the J-CMH@$CaCO_3$/T. (b) serum calcium concentration of different.

As shown in **Figure S8a**, it is noticed that Ca^{2+} release kinetics of the J-CMH@$CaCO_3$/T was biphasic, the initial rapid release of Ca^{2+} lasted for 50 s, and was followed by a second slow releasing phase until the concentration peak was reached at 175 s. To clarify the safety of hydrogel spheres applied on in vivo experiments, the serum calcium concentration of the J-CMH@$CaCO_3$/T was detected. Serum calcium concentration of the J-CMH@$CaCO_3$/T presented in **Figure S8b** was similar to that of blank, medical gauze and the CMH, indicating that Ca^{2+} release of the J-CMH@$CaCO_3$/T had no effect on the blood calcium levels of rabbits. The released Ca^{2+} from the J-CMH@$CaCO_3$/T was trapped by blood clots after activating the hemostasis cascade. Thus, the released Ca^{2+} would not enter the blood circulation and induce thrombosis or even death.

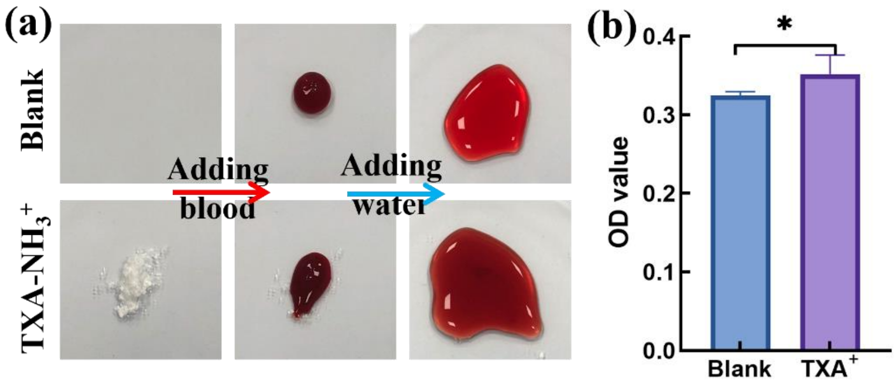

Figure S9. (a) Optical images showing blood coagulation induced by different samples. (b) A quantitative analysis of BCI.

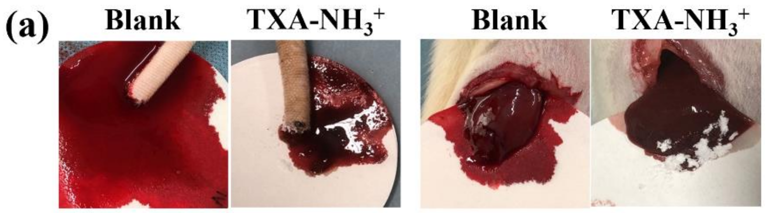

(b) **Tail** **Liver**

Name	Tail amputation		Liver injury	
	Time (s)	Loss (g)	Time (s)	Loss (g)
**Blank**	236±19	2.60±0.86	215±5	1.35±0.08
**$TXA\text{-}NH_3^+$**	290±8	2.35±0.17	172±15	1.13±0.09

Figure S10. (a) Images of hemostasis in tail amputation and liver bleeding model after depositing different samples. (b) Quantitative results of lost blood from bleeding tail and liver treated with different samples.

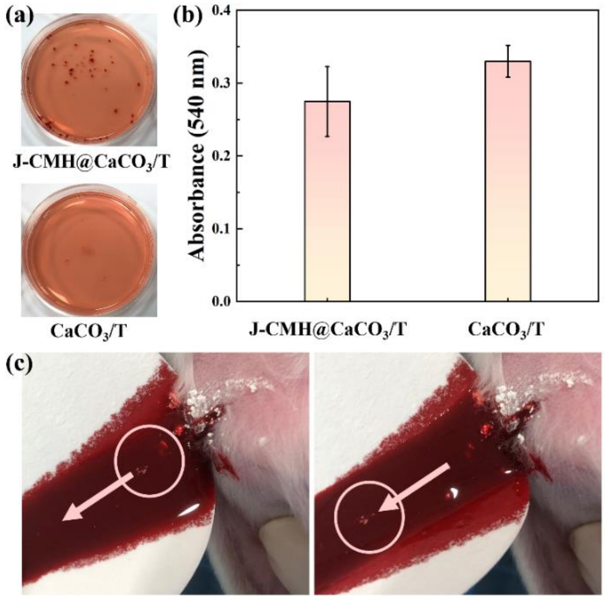

Figure S11. Photographs (a) and hemoglobin absorbance (b) of J-CMH@$CaCO_3$/T and $CaCO_3$/T. (c) Images of hemostasis in ear artery bleeding model of rabbits after depositing $CaCO_3$/T.

To investigate the hemostatic assay of $CaCO_3$ powder without hydrogel spheres, we further constructed *in vitro* and *in vivo* blood clotting experiments by adding $CaCO_3$/T (mixture of $CaCO_3$ powder and TXA-NH_3^+) to the whole blood or applying to the bleeding wound. As shown in **Figure S11a**, the blood diffusion in water treated with J-CMH@$CaCO_3$/T and $CaCO_3$/T was recorded by photographs. The J-CMH@$CaCO_3$/T showed a lower absorbance compared to the $CaCO_3$/T group presented in **Figure S11b**, demonstrating a better hemostatic performance *in vitro*. The J-CMH@$CaCO_3$/T hydrogel spheres could absorb the blood and concentrate red blood cells and platelets, allowing $CaCO_3$ powder fully contacted with the TXA-NH_3^+ and then released Ca^{2+} to take charge of the hemostatic process. However, when the $CaCO_3$/T contacted with blood, the mixed powder was dispersed into the different areas of blood immediately, which detracted $CaCO_3$ powder to release Ca^{2+}. *In vivo* hemostasis of the $CaCO_3$/T was investigated by the rabbits-ear artery model. The hemostatic process was recorded by photographs **(Figure S11c)**. The ear artery bleeding model had a massive hemorrhage. Obviously, the $CaCO_3$/T could not passively drift with the strong blood flow from wounds, leading to unsatisfactory

bleeding control. Thus, the introduction of the hydrogel spheres was of great significance in hemostasis.

References

1. Huang, Y.; Zhao, X.; Zhang, Z.; Liang, Y.; Yin, Z.; Chen, B.; Bai, L.; Han, Y.; Guo, B., Degradable Gelatin-Based IPN Cryogel Hemostat for Rapidly Stopping Deep Noncompressible Hemorrhage and Simultaneously Improving Wound Healing. *Chemistry of Materials* **2020,** *32* (15), 6595-6610.
2. Zou, F. X.; Wang, Y. S.; Zheng, Y. D.; Xie, Y. J.; Zhang, H.; Chen, J. S.; Hussain, M. I.; Meng, H. Y.; Peng, J., A novel bioactive polyurethane with controlled degradation and L-Arg release used as strong adhesive tissue patch for hemostasis and promoting wound healing. *Bioactive Materials* **2022,** *17*, 471-487.
3. Yang, E. R.; Hou, W.; Liu, K.; Yang, H.; Wei, W. Y.; Kang, H. F.; Dai, H. L., A multifunctional chitosan hydrogel dressing for liver hemostasis and infected wound healing. *Carbohydrate Polymers* **2022,** *291.*
4. Zou, C. Y.; Lei, X. X.; Hu, J. J.; Jiang, Y. L.; Li, Q. J.; Song, Y. T.; Zhang, Q. Y.; Li-Ling, J.; Xie, H. Q., Multi-crosslinking hydrogels with robust bio-adhesion and pro-coagulant activity for first-aid hemostasis and infected wound healing. *Bioactive Materials* **2022,** *16*, 388-402.
5. Zhang, H. J.; Zhang, J. H.; Peng, X.; Li, Z.; Bai, W. J.; Wang, T. Y.; Gu, Z. P.; Li, Y. W., Smart Internal Bio-Glues. *Advanced Science* **2022,** *9* (27).
6. Zhao, X.; Liang, Y. P.; Guo, B. L.; Yin, Z. H.; Zhu, D.; Han, Y., Injectable dry cryogels with excellent blood-sucking expansion and blood clotting to cease hemorrhage for lethal deep-wounds, coagulopathy and tissue regeneration. *Chemical Engineering Journal* **2021,** *403.*
7. Cheng, H.; Shi, W.; Feng, L.; Bao, J.; Chen, Q.; Zhao, W.; Zhao, C., Facile and green approach towards biomass-derived hydrogel powders with hierarchical micro-nanostructures for ultrafast hemostasis. *Journal of Materials Chemistry B* **2021,** *9* (33), 6678-6690.
8. Smith, J. R.; Bolton, E. R.; Dwinell, M. R., The Rat: A Model Used in Biomedical Research. In *Rat Genomics*, Hayman, G. T.; Smith, J. R.; Dwinell, M. R.; Shimoyama, M., Eds. Springer New York: New York, NY, 2019; pp 1-41.
9. Ahmadian, Z.; Correia, A.; Hasany, M.; Figueiredo, P.; Dobakhti, F.; Eskandari, M. R.; Hosseini, S. H.; Abiri, R.; Khorshid, S.; Hirvonen, J.; Santos, H. A.; Shahbazi, M.-A., A Hydrogen-Bonded Extracellular Matrix-Mimicking Bactericidal Hydrogel with Radical Scavenging and Hemostatic Function for pH-Responsive Wound Healing Acceleration. *Advanced Healthcare Materials* **2021,** *10* (3).
